# Integrating serological and genetic data to quantify cross-species transmission: brucellosis as a case study

**DOI:** 10.1017/S0031182016000044

**Published:** 2016-03-03

**Authors:** MAFALDA VIANA, GABRIEL M. SHIRIMA, KUNDA S. JOHN, JULIE FITZPATRICK, RUDOVICK R. KAZWALA, JORAM J. BUZA, SARAH CLEAVELAND, DANIEL T. HAYDON, JO E. B. HALLIDAY

**Affiliations:** 1 Boyd Orr Centre for Population and Ecosystem Health, Institute of Biodiversity, Animal Health and Comparative Medicine, College of Medical Veterinary and Life Sciences, University of Glasgow, Glasgow G12 8QQ, UK; 2 Nelson Mandela African Institution of Science and Technology, School of Life Sciences and Bioengineering, Arusha, Tanzania; 3 National Institute of Medical Research, PO Box 9653, 11101 Dar es Salaam, Tanzania; 4 Moredun Research Institute, Pentlands Science Park. Penicuik, Midlothian EH26 0PZ, UK; 5 Department of Veterinary Medicine and Public Health, Sokoine University of Agriculture, P.O. Box 3021, Morogoro, Tanzania

**Keywords:** data integration, serology, brucellosis, genetics, epidemiological modelling, Bayesian modelling, state-space models

## Abstract

Epidemiological data are often fragmented, partial, and/or ambiguous and unable to yield the desired level of understanding of infectious disease dynamics to adequately inform control measures. Here, we show how the information contained in widely available serology data can be enhanced by integration with less common type-specific data, to improve the understanding of the transmission dynamics of complex multi-species pathogens and host communities. Using brucellosis in northern Tanzania as a case study, we developed a latent process model based on serology data obtained from the field, to reconstruct *Brucella* transmission dynamics. We were able to identify sheep and goats as a more likely source of human and animal infection than cattle; however, the highly cross-reactive nature of *Brucella* spp. meant that it was not possible to determine which *Brucella* species (*B. abortus* or *B. melitensis*) is responsible for human infection. We extended our model to integrate simulated serology and typing data, and show that although serology alone can identify the host source of human infection under certain restrictive conditions, the integration of even small amounts (5%) of typing data can improve understanding of complex epidemiological dynamics. We show that data integration will often be essential when more than one pathogen is present and when the distinction between exposed and infectious individuals is not clear from serology data. With increasing epidemiological complexity, serology data become less informative. However, we show how this weakness can be mitigated by integrating such data with typing data, thereby enhancing the inference from these data and improving understanding of the underlying dynamics.

## INTRODUCTION

It is a regrettable but ubiquitous state of affairs that we are unable to directly observe those aspects of the ecology of a pathogen that are most informative of its epidemiological dynamics. For example, we may be able to observe how the prevalence of a disease changes over space and time, but not the underlying transmission processes that give rise to these changes. We may also be able to observe the presence of clinical disease, but not when a host became infectious. Similarly, the identification of antibodies through serological assays can reveal past exposure of a host to a pathogen but inferring precisely when this exposure occurred and what it implies about infectiousness can be challenging.

There is an increasing availability of large-scale, cross-sectional and longitudinal serology datasets from human, livestock and wildlife systems around the world, as the technological requirements necessary to generate serological data are low and evidence of exposure can persist. The analysis of serological data is a primary methodology for investigating the prevalence and transmission dynamics of infectious diseases. However, in addition to inferring the timing of exposure, there are several factors that complicate the epidemiological insight that can be gained from it. For example, serological data are generated by tests with imperfect sensitivity and specificity. In addition, the antibodies detected may also have been generated in response to a number of closely related pathogens that may be circulating independently of each other (known as cross-reactivity). Cut-off thresholds for distinguishing between exposed and unexposed individuals are often ambiguous, and antibodies may decline with time after the peak immune response that follows exposure (Gilbert *et al.* 2013). These factors all create challenges for the interpretation of, and inference from, serological data.

How should epidemiologists proceed when confronted by data that are only weakly informative of the dynamics that we so urgently need to understand? We could simply reject such ‘weak’ data and organize the collection of more informative data. However, this is not always possible and is likely to be time consuming, expensive, wasteful, and has potential ethical and welfare implications in terms of unnecessary animal handling and sampling. Furthermore, as a reflection of the immune systems ‘memory’ of historical exposure, serological data are information rich. Rather than reject these imperfect data we should instead develop more sophisticated analyses to interrogate the data we have and extract the information they contain (Jones *et al.* 2009, 2012; Norris *et al.* 2009).

The most effective response to these analytical challenges will involve a pragmatic combination of approaches, including the development of techniques that allow better use of serological data through integration with additional data relating to other observable aspects of the system. While each of these data types may be only weakly informative on its own, considered together they can strengthen each other (Strelioff *et al.* 2013; Viana *et al.* 2014). The effective synthesis of available data is an intuitive and increasingly recognized approach in many scientific disciplines (Searls, 2005). However, integration of multiple different types of data to conduct robust analysis is a challenge in itself that requires significant methodological development.

Current Bayesian statistical methods facilitate this approach (Gelman, 2004) and are becoming increasingly popular among epidemiologists (Basanez *et al.* 2004; Broemeling, 2014). For example, networks of ‘who-infected-who’, known as transmission trees (Morelli *et al.* 2012; Jombart *et al.* 2014; Mollentze *et al.* 2014), have been used to integrate epidemiological, genetic and transmission data from the outbreak of foot-and-mouth disease virus in the UK in 2001, and greatly enhanced understanding of the spread of the epidemic. By simultaneously analysing the time that farms were reported as infected, their geographic locations and the age of the oldest lesions found on livestock on each infected farm, it was possible to estimate a transmission tree (Haydon *et al.* 2003). The further inclusion of whole genome sequence data of the virus isolated from each farm substantially reduced the number of potentially plausible transmission trees (Cottam *et al.* 2008). Similarly, state-space models (Patterson *et al.* 2008; Holdo *et al.* 2009; Hooker *et al.* 2011; Viana *et al.* 2015) also known as hidden or latent process models, can enable inference of the unobserved (or hidden) epidemiological process behind the generation of observations. These have recently been used to integrate data on the age of the host, and the timing of vaccination campaigns with serology data to reconstruct the dynamics of Canine Distemper Virus outbreaks in populations of lions and dogs in and around the Serengeti National Park (Viana *et al.* 2015).

The overall aim of this paper is to explore how widely available serology data can be enhanced by integration with less common type-specific data, to improve the understanding of the transmission dynamics of complex multi-species pathogens and host communities. We focus on brucellosis as an example of such a system, but note that there are several important infectious diseases for which serology data are relatively common and type-specific data much rarer, for example, leptospirosis, foot-and-mouth disease and blue tongue.

### Case study: brucellosis in northern Tanzania

Brucellosis is a bacterial zoonosis that has a worldwide distribution, but human disease incidence is higher in low- and middle-income countries (Dean *et al.* 2012*b*). In humans, it causes non-specific febrile illness, debilitating symptoms, including joint and muscle pain, as well as more severe complications such as endocarditis and neurological symptoms (Dean *et al.* 2012*a*). Multiple animal species, including livestock, are affected by brucellosis which impacts on productivity through abortion, reduced reproductive efficiency and decreased milk production. Human infections are typically acquired through contact, ingestion or inhalation of bacteria shed by infected animals.

Brucellosis can be caused by one of several bacteria of the genus *Brucella.* The two species of greatest relevance for human and livestock health in northern Tanzania are *Brucella abortus*, which is typically thought to be associated with cattle, and *Brucella melitensis*, which is often reported in sheep and goat populations (hereafter combined and referred to as ‘caprids’) (World Health Organization *et al.* 2006). The main risk factor identified as the driver of animal and human infection is large animal population size (Kadohira *et al.* 1997; McDermott and Arimi, 2002). However, the known capacity for *B. abortus* and *B. melitensis* to be transmitted between cattle and caprids complicates the interpretation of the roles played by different host populations in the maintenance and transmission of this disease.

In sub-Saharan Africa, current efforts to develop control strategies are constrained by limited understanding of two fundamental features of brucellosis epidemiology: (1) which animal species are affected and act as the source of human infections; and (2) which *Brucella* species are maintained in which animal hosts? The distinction among epidemiological scenarios is crucial because vaccination of animals is currently the most effective method to control brucellosis (Godfroid *et al.* 2011), but vaccines are pathogen species and host-specific; i.e. for *B. abortus* vaccine for cattle and *B. melitensis* vaccine for caprids.

Most epidemiological data on brucellosis in sub-Saharan Africa are obtained through serological surveys. Brucellosis seroprevalence data are compound representations of the occurrence of the two *Brucella* species that cross-react with the test antigen (McGiven, 2013). A seropositive status is therefore an indication of exposure to *B. melitensis* and/or *B. abortus*. Genetic detection allows identification of the infecting species of *Brucella*, but this is likely to be successful only if sample collection occurs within the short-time window around either acute illness in people, or the time of parturition or abortion in animals. The considerable investment required to obtain large numbers of these samples makes such data extremely scarce.

**Fig. 1.**
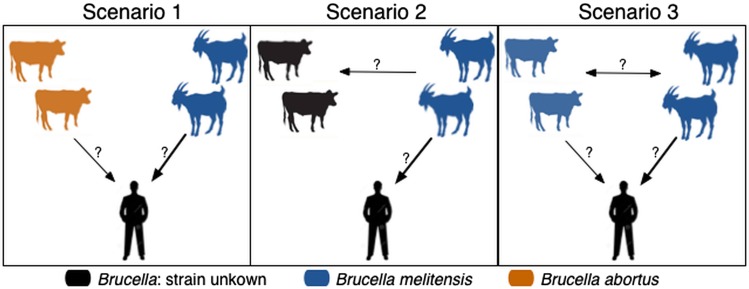
Plausible alternative epidemiological scenarios for inter-species transmission and sources of human *Brucella* infection. Arrows indicate the direction and magnitude of transmission. Question marks indicate that transmission occurs with an unknown magnitude (which will be estimated by our models). In Scenario 1, humans can be infected by caprids with *B. melitensis* and cattle with *B. abortus*; in Scenario 2 caprids with *B. melitensis* can transmit to humans and cattle but only caprids can transmit infection to humans; and in Scenario 3 both caprids and cattle with *B. melitensis* can infect humans.

**Fig. 2.**
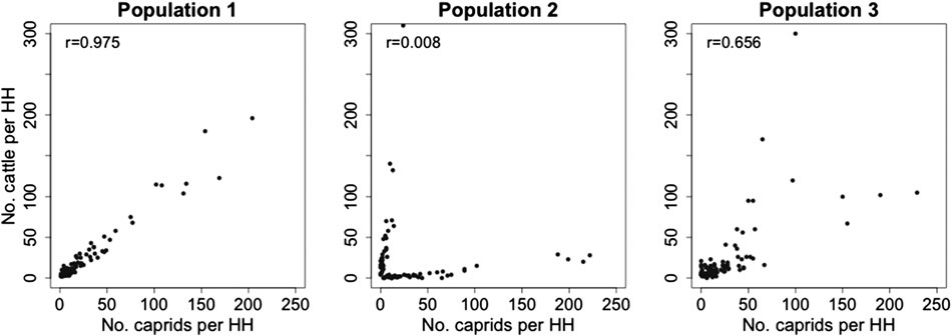
Population structures used in simulations. In population structure 1 there is a positive correlation between cattle and caprid numbers in each household (HH). In population structure 2 there is clear segregation and each household has mostly cattle or mostly caprids. Population structure 3 shows an intermediate relationship with weak correlation of cattle and caprid numbers and represents the structure of the real sampled population from northern Tanzania.

Figure 1 depicts three of several possible scenarios of brucellosis transmission in northern Tanzania, which we will use to illustrate challenges and possible solutions to better understanding these complex epidemiological systems. The power to distinguish between these transmission scenarios likely depends on how the different hosts co-occur. For example, cattle and caprids can be highly positively correlated (e.g. sampled households with more cattle have more caprids), clearly segregated (e.g. sampled households contain mostly one host species) or weakly correlated. These possible population structures are demonstrated in Fig. 2.

The aim of this paper is to answer two fundamental questions:

(1) When can analysis of serology alone identify the source of human *Brucella* infection?
(2) When does the integration of realistically sparse genetic-typing data allow identification of the appropriate source of infection when serology alone does not?

To address these questions, we first develop a method to determine the animal source of human *Brucella* spp. infection and quantify cross-species transmission between cattle and caprids using field serology data from our *Brucella* case study. Second, we use simulated data to explore how different epidemiological scenarios and population structures might impact on the power of serological data to reveal similar transmission patterns to those observed in our data. Third, we simulate genetic type-specific data to augment the serological data and explore the circumstances under which this integration enhances serology data and provides enough analytical power to distinguish between the different transmission scenarios.

## MATERIALS AND METHODS

### Field serological survey

#### Data collection

**Fig. 3.**
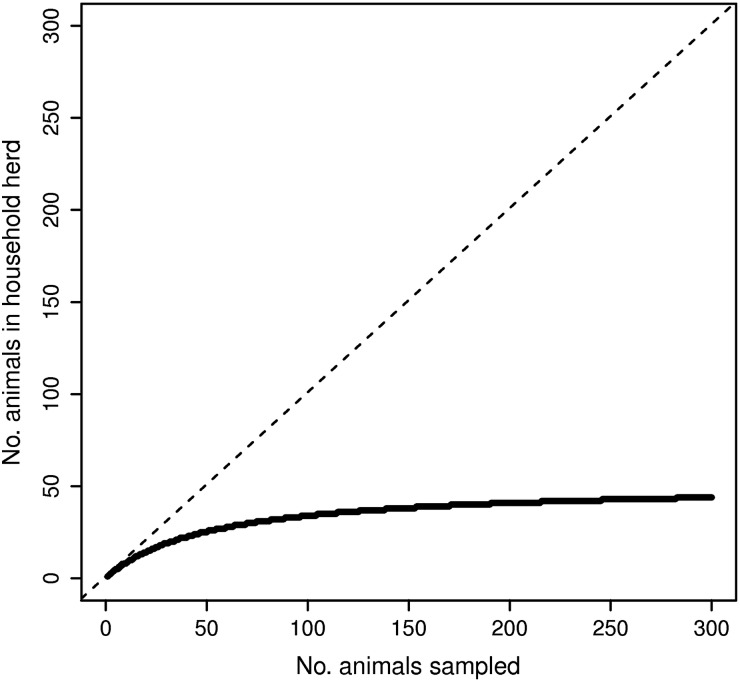
Serology sampling strategy. The continuous bold line shows the relationship between the number of animals present at each household and the number sampled. The dotted line shows the number of animals that would be sampled if all animals present were sampled.

The serological data on brucellosis were collected through a cross-sectional field study conducted in the Arusha and Manyara regions of northern Tanzania in 2002–2003. Within five districts in these regions, a multi-stage cluster sampling strategy was used to identify and randomize the selection of villages, sub-villages, ten cell units and livestock keeping households. Data collection at the 86 selected households included blood sample collection from randomly selected sheep and goats (combined as caprids), cattle and humans. The number of cattle and/or caprids sampled at each household was based on the number required to estimate the within-herd prevalence given the size of the household herd. This was calculated for an expected prevalence of 5%, with 80% power and 95% confidence (Martin *et al.* 1987). All humans present at the household were invited to participate in the study. Serum samples from all species were tested for antibodies against *Brucella* species at the Animal and Plant Health Agency (APHA) using a competitive ELISA (McGiven *et al.* 2003; Perrett *et al.* 2010). A cut-off of 60% inhibition was used to define sample status for all species tested. This dataset is a subset of data described previously (Shirima, 2005; Kunda, 2006). Figure 2c shows the number of cattle and caprids sampled in each household and Fig. 3a shows the seroprevalence of *Brucella* sp. in humans, cattle and caprids in this dataset. No genetic-typing data of the *Brucella* species were available in this field study.

#### Generalized linear model (GLM) analysis

GLMs were used to examine the relationship between the seroprevalence of *Brucella* exposure in humans and the (crudely) estimated total numbers of seropositive cattle and caprids present at each household in the Tanzanian field dataset. A GLM was used instead of a GLMM because the inclusion of random effects (e.g. household and village) did not make a difference to the results, both in terms of significance and goodness-of-fit of the model (i.e. AIC difference was less than 2; Burnham and Anderson, 2002). The response variable was the proportion of humans at the household that tested seropositive, weighted by the number of humans sampled at the household. The covariates considered were estimates for the total numbers of seropositive cattle and caprids present at each household, which were calculated by multiplying the proportion seropositive in the sample by the total population size at the household for each animal group.

#### Bayesian serology model

A latent model was developed to quantify the contribution of different animal hosts to human infection probability from serology data. A detailed description of this model is provided in Supplementary information (SI). In essence, the model is composed of five interlinked binomial processes: the first two binomials estimate the cattle and caprid probability of being seropositive in the sampled population, the third and fourth binomials take these inferences from the sample to estimate the number of seropositive cattle and caprids in the whole population, and the fifth binomial process uses the population inferences to estimate the contribution of seropositive animals to the probability of human infection. One of the main advantages of this modelling approach is that it allows propagation of uncertainties from the inferences of the animal samples, to those of the whole animal population and finally to humans. We note that this is not a one-way propagation as the five binomials are estimated simultaneously and inform each other.

For each host [i.e. cattle *c*, caprids (sheep and goats) *s* and humans *h*], a binomial model (equivalent to a Bernoulli process) is implemented to describe the observation process of each individual serology test result. The likelihood of the data from an individual being classified as seropositive was based on the serological test data and a probability of misclassification. This probability of misclassification (i.e. whether the serology assay generates false positives or negatives (further details in the SI) typically incorporates pre-existing information about the performance of the test being used. For simplicity and because there is no misclassification in the simulated data, this was always set to 0. These binomials are ultimately defined by a probability of an individual testing seropositive in a household. At the household level, these are linearized through a logit transformation and described through a linear predictor (lnSer). For humans, this linear predictor is described below. For cattle (lnSer_c_) and caprids (lnSer_s_) these predictors are described as a function of the number of cattle [Ncattle(*i*)] and caprids [Ncaprids(*i*)] in each household (*i*):
1
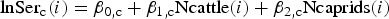

2
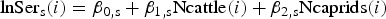

where *β*_0,c_ and *β*_0,s_ correspond to the intercepts, *β*_1,c_ and *β*_2,c_, and *β*_1,s_ and *β*_2,s_, correspond to the coefficients governing the effect of the number of cattle and caprids on the probability of infection in cattle and caprids, respectively. The unobserved total numbers of infected cattle and caprids per household [*Y*_c_(*i*) and *Y*_s_(*i*)] are then estimated through a Binomial process using the infection probability estimated from the sampled population (pSer, which corresponds to the inverse logit of the predictor lnSer) and data on the total herd/flock size at each household:
3
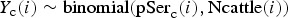

4
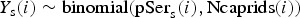


The estimated total numbers of positive animals [*Y*_c_(*i*) and *Y*_s_(*i*)] are then used as covariates in the logit linear predictor [lnSer_h_(*i*)] of the probability of human infection:
5
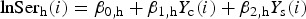

where *β*_0,h_ corresponds to the intercept, *β*_1,h_ and *β*_2,h_ correspond to the coefficients governing the effect of the number of infected cattle and caprids on the probability of human infection.

Further details of the model, including the prior distributions allocated to each coefficient and the model code for implementation in JAGS, are provided in the SI.

This model only used serology data and was implemented with the field survey data to estimate the human source of infection in the northern Tanzanian setting. This serology only model was also implemented with simulated datasets (see the subsequent section) to address our specific aim 1 and explore how different epidemiological scenarios and population structures might impact on the inferences made from serological data.

### Simulation study

#### Simulation of serology & genetic typing data

We simulated datasets to illustrate the three alternative epidemiological scenarios of *Brucella* transmission (Fig. 1) within three distinct population structures (Fig. 2). In epidemiological scenario 1, cattle were infected with *B. abortus* only and caprids with *B. melitensis* only. Humans could acquire *Brucella* infection from both livestock populations, but the contribution of *B. melitensis*-positive caprids to human infection probability was twice as large as that of *B. abortus*-positive cattle. See Table S3 for simulation coefficient values. In this case, genetic-typing data were simulated only for *B. abortus*-positive cattle and *B. melitensis*-positive caprids (see below for details of the sampling mechanisms used for simulations). In epidemiological scenario 2, only *B. melitensis* was present. Here, cattle could be exposed to infection from caprids and seroconvert but humans could only acquire infection from caprids. This illustrates a situation where cattle do not shed the pathogen and no genetic-typing data are available. In epidemiological scenario 3, only *B. melitensis* was present but both caprids and cattle were infected. Genetic-typing data were simulated for *B. melitensis*-positive cattle and caprids and the probability of obtaining typing data from seropositive individuals in these two groups was equal. In this scenario, humans acquire infection from both hosts, but the contribution of *B. melitensis*-positive caprids to human infection probability is twice as large as the contribution of *B. melitensis*-positive cattle. We note that there is no genetic-typing data for humans, and that in all simulations the only data available for humans are serological test results. Scenarios where only *B. abortus* is present were not included but these would be functionally equivalent to the *B. melitensis* only scenarios explored in scenarios 2 and 3.

Each of the epidemiological scenarios was simulated for the three distinct population structures in Fig. 2. The simulated population structures included 86 households and 428 humans (identical to the household and human numbers in the field dataset). The total size of the livestock population in each household (cattle + caprids) was also as observed in the field study, but the ratio of cattle:caprids was altered for each population structure. In population structure 1, the total animal population was divided between cattle and caprids (with a probability of 0·5 of being a cow *vs* caprid), leading to a strong positive correlation (measured by the Pearson's correlation coefficient, *r*) between cattle and caprid numbers in each household (*r* = 0·975). In population structure 2, there is clear segregation of the cattle and caprid populations such that each household had either 90% cattle or 90% caprids (*r* = 0·08). In population structure 3, the number (and ratio) of cattle and caprids in each household was as observed in the field dataset (*r* = 0·656).

The models and parameters used to simulate the alternative datasets are based on the serology model presented above, and its extension that includes genetic-typing data presented below. In brief, animal infection probability was simulated as function of the size of the cattle and caprid populations at the household level. Human infection probability was simulated as a function of the number of animals in each of four possible infectious populations: *B. abortus* infected cattle, *B. abortus* infected caprids, *B. melitensis*-infected cattle and *B. melitensis*-infected caprids. The parameter values used for these simulations differed for the different epidemiological scenarios and the details of the values used are given in Table S3. The plausibility of the values used for the simulated datasets was ensured by keeping the number of households, the number of human and animals per household (explained above), and the prevalence values generated within realistic values (i.e. similar to those in the field dataset). Simulated prevalence values were below 10% for cattle and caprid populations and 16% for humans.

To simulate both genetic-typing and serological data, the *Brucella* species-specific (*B. melitensis* or *B. abortus*) infection status (infected or not) was simulated at the individual animal level. For simplicity, all genetically positive individuals were classified as seropositive, analogous to assuming that all animal infections would lead to a detectable and lasting antibody response. This is an oversimplification that assumes that there is no misclassification in serostatus and that only seropositive animals can be genetically positive. We deal with the latter issue in the model analyses by considering such samples (genetically typed positives from seronegative individuals) as missing data. This simulation strategy also meant that all seropositive animals were positive for *B. melitensis* and/or *B. abortus*, corresponding to an assumption that the *Brucella* species responsible for all seropositivity could be determined in all cases. The exception to this assumption is the genetic-typing data simulated for cattle in epidemiological scenario 2. Here, all cattle test negative for *B. melitensis* as they constitute a dead-end host and would not shed *Brucella*.

As complete sampling of populations is rarely achievable in field studies, we sampled individuals from these simulated datasets using a rationale analogous to the sampling strategy used in the original field study; i.e. the sample size required to estimate prevalence of 5% with 95% confidence and 90% precision (Dohoo *et al.* 2003). Figure 3 illustrates the number of animals sampled from the range of population sizes. Only the data obtained from these sampled animals were considered in the model analyses.

While the determination of the serostatus of an individual is relatively straightforward, the apparently intermittent or temporally variable nature of *Brucella* shedding by the infected animals (World Health Organization *et al.* 2006; Ebrahimi *et al.* 2014), the potential for animals to seroconvert and recover (to a genetically negative but seropositive status) and relatively low diagnostic sensitivity of PCR-based techniques for the direct detection of *Brucella*, all ensure that the genetic identity of the infecting *Brucella* species will only ever be obtainable for a small subset of previously infected animals. To represent the sparseness of available genetic-typing data and explore the amount of typing data required to effectively identify the host and pathogen species that pose the greatest threat to human populations, we used different proportions of the genetic-typing data available for the seropositive animals sampled, i.e. 50, 10 and 5% (e.g. for the 5% situation, genetic-typing data were only available for 5% of the seropositive animals in the sampled population).

#### Model extension to integrate serology with genetics

In order to integrate genetic-typing data, we extended the serology model described above to include the influence of different pathogen and animal host combinations upon the probability of human infection. In this extended model, we replace lnSer_h_(*i*) by lnType_h_(*i*) and define this linear predictor as:
6
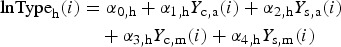

where *α*_0,h_ corresponds to the intercept, *α*_1,h_ and *α*_2,h_ correspond to the coefficients governing the effect of the number of *B. abortus* infected cattle and caprids (*Y*_c,a_ and *Y*_s,a_), respectively, and *α*_3,h_ and *α*_4,h_ correspond to the coefficients governing the effect of the number of *B. melitensis* infected cattle and caprids (*Y*_c,m_ and *Y*_s,m_), respectively, on the probability of human infection. The main difference between the extended model and the serology only model is in the way the ‘true’ number of infected cattle and caprids in each household are estimated [i.e. four *Y*'s from equation (6) compared with the two *Y*'s from equation (5)]. In this extended model, the number of *B. abortus* and *B. melitensis-*infected cattle and caprids were estimated from Binomial processes in which the probability of being infected with a pathogen/host combination (pType) is weighted by the probability of the host being seropositive (pSer), which was estimated from the serology only model. It is in this step that the integration between serology and genetic-typing data occurs:
7
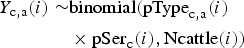

8
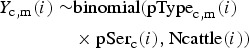

9
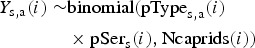

10
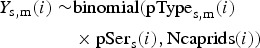


The remaining components of this model are equivalent to those of the serology only model. However, the probability of being genetically positive (pType) comes from the binomial likelihood of the data [e.g. for *B. abortus* in cattle ~binomial(pType_c,a_(*i*), Ncattle(*i*))], rather than being a Bernoulli trial, as we assume that the misclassification associated with genetic typing is negligible (e.g. there are no incorrectly typed individuals by PCR-based diagnostics). This probability pType is finally linearized (lnType) through a logit transformation and described as a function of the number of animals in the household. For cattle with *B. abortus* [lnType_c,a_(*i*)]:
11
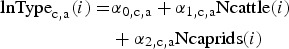


The parameter *α*_0,c,a_ corresponds to the intercept, *α*_1,c,a_ and *α*_2,c,a_ correspond to the coefficients governing the effect of the number of cattle and caprids in the household. Further details of the model, including the full linear predictors for the remaining sources of infection (e.g. *B. abortus* infected caprids, *B. melitensis* infected cattle and *B. melitensis*-infected caprids), are given in the SI.

Although the model is a stochastic one, the model formulation matches the process of data simulation. Consequently, it should allow us to retrieve the coefficient values used to generate the contributions of the different infected animal populations to the probability of human infection. In addition, to verifying that we could retrieve the coefficient values used for simulations, we further determine the goodness-of-fit by evaluating its fit to the data, confirming that we can recover the generated animal population sizes and prevalence values, and by checking convergence of the model. Further details are given in the SI.

## RESULTS

### Brucella field survey: GLM analysis

The GLM analysis revealed no statistically significant relationships between the human seroprevalence and the predicted number of seropositive caprids or cattle at the household. The coefficient estimates and standard errors (s.e.) obtained in the model are given in Table 1.

**Table 1.** Summary of the GLM analysis examining the relationship between human *Brucella* seroprevalence and the seropositive population size of caprids and cattle at each household in the Tanzanian field dataset

Variable	Coeff.	s.e.	*P*	Odds ratio	95% CI
Predicted no. positive caprids	0·016	0·017	0·35	1·02	0·98–1·051
Predicted no. positive cattle	−0·018	0·029	0·54	0·98	0·93–1·039

### Serology model applied to the field data

**Fig. 4.**
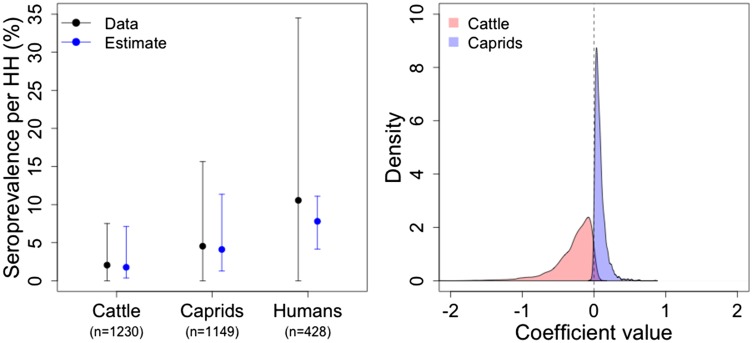
Results from the serology model on the *Brucella* field survey data. The left panel shows the raw mean seroprevalence per household, per species (with associated s.d.; black) and the equivalent model estimated means (with associated 95% credible intervals; blue). The right panel shows the posterior distributions of the coefficients governing the contribution of cattle (*β*_1,h_ in red) and caprids (*β*_2,h_ in blue) to the probability of human infection in northern Tanzania.

The results of the serology model analysis of the field study data suggest that the model was appropriate to describe animal and human *Brucella* infection. The similarity of the mean seroprevalence values per household of each host population calculated from the data (Fig. 4 black bars) and those estimated from our serology model (Fig. 4 blue bars) is an indication of good model fit. Later we also show that we can retrieve all the coefficients used in the simulations with our model. Although the mean seroprevalence estimated for humans was somewhat underestimated by the model, the estimated credible intervals fall within the standard deviation (s.d.) of the data, which has a large variability.

Caprids were identified by our model as the main source of human infection in northern Tanzania field data. This is shown in Fig. 4 (right panel). The posterior distribution of the coefficient governing the contribution of *Brucella* seropositive caprids to human infection probability [blue, i.e. *β*_2,h_ in equation (5)] is 95% above zero. In comparison the distribution for seropositive cattle [red, i.e. *β*_1,h_ in equation (5)] is below or close to zero (Fig. 4, right panel).

**Fig. 5.**
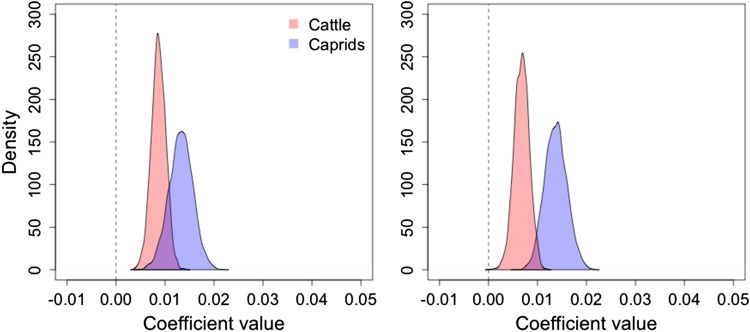
Model estimates for the influence of animal population size on infection probability of cattle (*β*_1,c_ & *β*_2,c_, left panel) and caprids (*β*_1,s_ & *β*_2,s_, right panel).

Our results showed increased infection probability in larger livestock populations. All the coefficients governing the contribution of cattle (Fig. 5, red) and caprid (Fig. 5, blue) household population size to the probability of infection in cattle [*β*'s from equation (1); Fig. 5 left panel] and caprids [*β*'s from equation (2); Fig. 5 right panel] were positive (i.e. at least 95% of the credible interval of each posterior distribution is greater than zero). However, the caprid population size (blue) seems to have a greater positive influence on both caprid and cattle infection probability than cattle population size (red).

### Simulation study

**Fig. 6.**
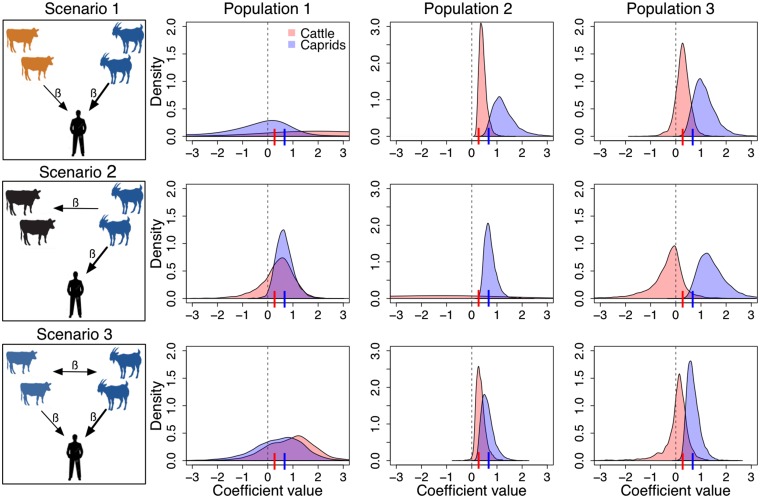
Posterior distributions of the coefficients governing the effect of *Brucella*-seropositive cattle (*β*_1,h_ in red) and caprids (*β*_2,h_ in blue) on the probability of human infection. These posteriors were obtained from the serology only model applied to each combination of the epidemiological scenarios and population structures used for simulations. Small vertical lines on the *x*-axes correspond to the coefficient values used for simulation.

The results of the serology only analysis of the simulated data (Fig. 6) show that the ability of our model to accurately quantify the contribution of different livestock populations to human infection probability depends on the epidemiological scenario and the population structure of the animal hosts involved. Figure 6 shows the posterior distributions of the coefficients governing the influence of seropositive cattle (red) and caprids (blue) upon the probability of human infection [*β*'s in equation (5)] from each combination of the different epidemiological scenarios (rows) and population structures (columns). If the coefficient value used in the simulations (indicated as a small vertical bar on the *x*-axis) falls within the respective posterior distribution, and the 95% credible intervals are above zero, it suggests that the model accurately quantified the contribution to human infection probability. However, if the 95% credible intervals cross zero, it suggests that the host population associated with that coefficient was not found to have a significant contribution to human infection. Nonetheless, only when the posterior is flat (typically not visible in our plots), or 95% below zero can we be certain that the host does not contribute to human infection.

Figure 6 suggests that in positively correlated host populations (e.g. Population structure 1) it is difficult to identify the main host source of human infection from serology alone, as the posteriors for both caprids and cattle are similar, highly variable and their 95% credible intervals crosses zero. In epidemiological scenario 2 the contribution of caprids was accurately quantified but cattle are still (wrongly) implicated in human infection in some cases. In contrast, in the presence of an uncorrelated host population (Population structure 2), serology data alone seem to be sufficient to identify the host sources and quantify their contribution to human infection, in all epidemiological scenarios investigated. For moderately correlated host populations (Population structure 3) our results show that the main source of human infection (i.e. caprids in all cases) is accurately identified and its contribution to human infection is quantified reasonably well (although sometimes the mean is slightly overestimated). However, the contribution of cattle to human infection is only accurately quantified in epidemiological scenario 1. In epidemiological scenarios 2 and 3, the 95% credible intervals of the posterior distributions governing the effect of cattle on human infection (red) cross zero, but their large percentage above zero are sufficient to consider them as potential sources. However, while cattle are correctly identified as a potential contributor to human infection in epidemiological scenario 3 (but with the wrong magnitude of contribution), in epidemiological scenario 2 cattle should not have been identified at all.

**Fig. 7.**
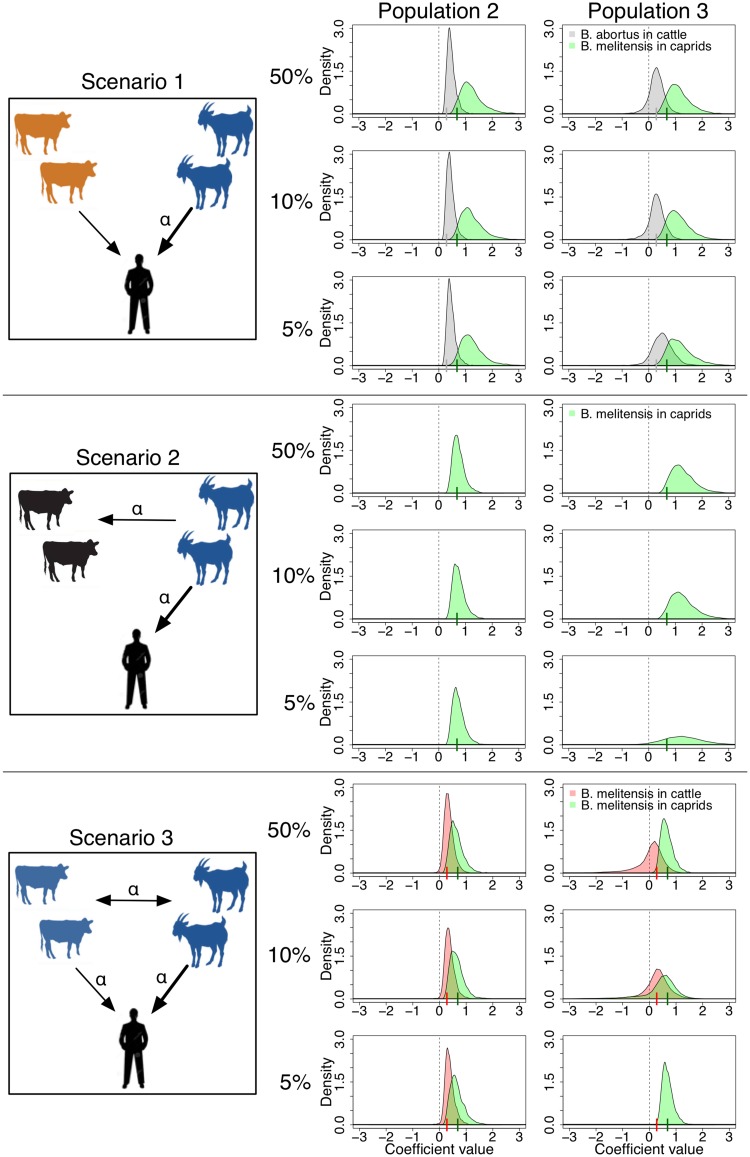
Posterior distributions for the coefficients describing the contributions of different infected animal populations to the probability of human infection from the model integrating genetic and serology data (*α*_1,h_ in green, *α*_3,h_ in red and *α*_4,h_ in grey), with decreasing levels of genetic-typing data (50% in top row, 10% in middle row and 5% in bottom row within each epidemiological scenario). Small vertical lines on the *x*-axes correspond to the coefficient values used for simulation.

The results of the extended model implemented with 50, 10 or 5% of genetic-typing data are shown in Fig. 7. Given the low resolution achieved by the serology data alone for population 1, results for this population are not included in the figure. The posterior distributions in Fig. 7 correspond to the estimated coefficients governing the influence of *B. abortus* and *B. melitensis*-positive cattle (grey and red, respectively) and *B. melitensis* in caprids (green) upon the probability of human infection [i.e. *α*'s in equation (6)] for each combination of epidemiological scenario (rows) and population structure (columns). We note that *B. abortus* in caprids is never visible as this pathogen–host combination never occurs, and hence its posterior distribution is null. This figure shows that even a small amount of genetic-typing data (e.g. 5%) is enough to empower serology data to distinguish the pathogen species being transmitted by the different hosts (see change in colour of the posterior distributions from Figs 6 and 7).

The integration of genetic-typing data with serology data also enables effective discrimination between infectious and exposed hosts. This is visible in the results of the extended model for epidemiological scenario 2, population structure 3, where the posterior distribution governing the apparent contribution of positive cattle to human infection probability (simulated with an effectively zero coefficient value) is no longer visible (as it was for the serology model, see red in Fig. 6).

The estimated host contributions from the extended model with 50% genetic-typing data are similar to those from the serology only model. However, as we decrease the amount of data to 10 and 5% the uncertainty in the coefficient estimates increases (as seen by the increasingly wide posterior distributions in Fig. 7). In examples such as epidemiological scenario 3 and population structure 3, we may lose completely the ability to identify a particular source of infection (see flattening of red posterior distribution for 5% of genetic-typing data). The amount of genetic-typing data necessary to add value to serology data depends on the population structure and epidemiological scenario. However, our results suggest that a sample of genetic-typing data from 5 to 10% of the serology samples may be sufficient to achieve the epidemiological insights that can be gained from the integration of these types of data.

We note that our model sometimes overestimates the coefficient value governing the influence of caprids or *B. melitensis*-positive caprids in human infection (see median of posterior distributions compared with simulated values). Nonetheless, these simulated values fall within the posterior distribution, which indicates that the model accurately describes the data but may require further information to reduce uncertainty.

## DISCUSSION

To effectively control infectious diseases, we must clearly identify the infecting pathogen(s) and distinguish between multiple possible epidemiological transmission scenarios. This need provided the motivation to explore the circumstances under which formal integration of data can improve our understanding of the disease dynamics. We use brucellosis in northern Tanzania as a case study, to develop a modelling framework that enables the accurate identification of the host and pathogen-specific source(s) of human infection, and show that integration of multiple types of data is a powerful technique. The value of this approach is most clear under two circumstances: (1) when more than one pathogen is present and serology data give a compound representation of the presence of multiple pathogens; and (2) the distinction between exposed and infectious individuals is not clear from serology data.

Brucellosis is transmitted to humans by animals. Animal vaccines exist but are host and pathogen species-specific, and both *B. abortus* and *B. melitensis* species have been isolated in Tanzania (Bouley *et al.* 2012; Mathew *et al.* 2015). This makes clear understanding of the multi-host infection dynamics critical, and without using the data integration approaches developed here we risk selecting inappropriate control options for brucellosis. The results of this study show that the animal population that constitutes the source of human *Brucella* infection can often be accurately identified with serology data alone. However, integration of genetic-typing data is essential to distinguish which pathogen generates the exposures observed. For example, the results shown in Fig. 7 (but not Fig. 6) for epidemiological scenarios 1 and 3 have quite different implications for the control measures that might be applied in these two situations. In both cases, caprids and cattle contribute with similar magnitudes to human infection. However, integration of genetic-typing data enables distinction of the contribution of *B. abortus-*positive cattle in scenario 1 from the *B. melitensis*-positive cattle in scenario 3. Second, in epidemiological scenario 2, cattle can be exposed to *B. melitensis* transmitted from caprids, but are not infectious to humans. In contrast, in epidemiological scenario 3 both caprids and cattle can transmit infection to humans. The difference in the roles played by the cattle in these two scenarios is fundamental, yet it is very difficult to discern using serological data only and the model can only do this in the more segregated populations. Despite the precision of the estimated coefficients decreasing with decreasing amounts of typing data, integration of even a small amount of genetic-typing data (e.g. 5%) enables the model to distinguish these two situations, so that it does not estimate a contribution of cattle to human infection probability in any of the scenario 2 datasets.

The improvement brought by the integration and the amount of genetic-typing data is influenced by both population structure and epidemiological scenario. The analyses of the simulated datasets indicate that when two host populations are clearly segregated, the analysis of serology data alone is sufficient to accurately identify the host source(s) of human *Brucella* infection under all of the epidemiological scenarios considered. However, with increasing levels of correlation between the two host populations it is increasingly difficult to identify the source of human infection from serology alone. Integration of genetic-typing data becomes more valuable in these more complex scenarios. It is also possible that with higher forces of infection (e.g. larger coefficients), some of the effects would be easier to capture. However, we have deliberately used coefficients of biologically plausible magnitude for the *Brucella* case study.

The specific results of the case study, i.e. from the brucellosis field serology survey; are consistent with the transmission processes illustrated in the epidemiological scenario 2. For northern Tanzania, caprids were estimated to be the most likely source of human *Brucella* exposure and the size of the household caprid population is a key driver of infection probability in both animal hosts. Current understanding of the host–pathogen preferences of *Brucella* suggests that *B. melitensis* is the most likely pathogen to be transmitted from caprids to humans (World Health Organization *et al.* 2006). However, only integration of genetic-typing data can confirm this hypothesis. The negative coefficient estimated for the contribution of *Brucella*-positive cattle to human infection could indicate some kind of protective effect of owning increasing numbers of cattle upon human probability of *Brucella* exposure. Cattle ownership is linked to socioeconomic status in the study area, which may lead to indirect impacts upon *Brucella* exposure probability. A preference for consumption of cow milk could also mean that consumption of milk from caprids would become increasingly unlikely in households with more cattle. However, the real-world plausibility of these explanations requires further investigation. We also cannot rule out the possibility that the estimation of this apparent protective effect may arise from an influence of population structure (e.g. posterior for epidemiological scenario 3 & population structure 3 peaks at positive values but a large portion is seen also at negative values).

All seroprevalence values estimated by the model for the field dataset are well within the s.d. seen in the data. However, we note the slight underestimate of the mean seroprevalence per household in humans from the model estimate as compared with the observed data. The credible interval of the model estimate is also considerably narrower than the equivalent s.d. in the observed data. This underestimation of both prevalence and uncertainty could suggest that in addition to the risk posed by the presence of infected animals within the household environment, human infection could also be influenced, to a greater extent than is true for cattle and caprids themselves, by factors external to the household environment that are currently not included in the model. Plausible factors would include for example the consumption of milk from animals from other households within the same village, district or region.

The results implicating caprids as the main source of human *Brucella* infection were not revealed by a standard GLM analysis. This is likely due to a more appropriate handling of the uncertainty structure in the Bayesian model as compared with the GLM. This may be particularly important for the estimation of the main risk factor, i.e. seropositive animal population size. The Bayesian approach allows propagation of uncertainty from individual animal samples to human inferences in the whole population. While intuitively this should just lead to a higher uncertainty in the final estimates, because the processes defined for animals and humans are interlinked and their inference are made simultaneously, they can inform each other and empower individual inferences.

In addition to identifying sources of infection, the modelling framework implemented here can highlight other aspects of the underlying disease dynamics. Strong associations between human and animal *Brucella* seropositivity at the household level have been recorded previously in East African populations (Osoro *et al.* 2015). However, other studies have found no associations (Zolzaya *et al.* 2014) or positive correlations are identified but only at larger spatial scales (Bonfoh *et al.* 2012). Increased correlation between human and caprid seroprevalences is also seen when our dataset is aggregated at the village and district levels as compared with the correlation seen at the household level (Shirima, 2005). The question of the optimal spatial unit to consider is likely to vary between settings and depend to a large extent on livestock management and animal–human interaction practices. The degree to which household herds are managed as closed units or mixed with herds from neighbouring households or villages, and the degree to which humans interact with their own animals only or the wider population, either directly or indirectly (e.g. through milk consumption), will impact on the scales at which transmission occurs. Although different patterns of correlation will be seen in different settings, questions about the links between infection in different hosts and the spatial scale at which any links occur are consistent themes in the recent brucellosis literature and calls have been made for greater efforts to understand the complexities of brucellosis transmission at the animal–human interface at different scales (Zolzaya *et al.* 2014).

In this study, we used household as the spatial scale for the analysis but the models could be applied at different spatial scales, or alternatively extended to include more complex spatial structuring providing flexibility for application to a range of settings. For example, we may wish to add a random effect of village to the linear predictors to estimate how much variability in the mean prevalence is driven by a sample being collected in a specific village (or village-associated factors) instead of a household. Other options, such as a full nested hierarchical model (e.g. household within villages, within regions) may also be desirable and are straightforward extensions to our model. The underestimation of the variance in the human seroprevalence at household level may reflect the absence of extra-household influences (associated with spatial effects mentioned above) on human infection probability in our model. As the network of the trade of dairy products become increasingly complex and involves more actors across greater spatial scales, the spatial scales that are important for understanding human brucellosis infection risk may increasingly become distinct from the scales at which transmission between cattle and caprids occurs. Other useful extensions to this modelling approach would be the inclusion of a formal distinction between the various forms of missing values (e.g. non-typed animals *vs* typed but undetected pathogen, or sensitivity/specificity in the typing data) and improvements of the observational process to include estimation of the timing of exposure (Viana *et al*. 2015).

Here we focused on a case study of *Brucella* in northern Tanzania; however, our findings and challenges are applicable to many systems, in which there is considerable need to clearly identify the infecting pathogen(s) that impact on human health (as well as livestock productivity) and to distinguish between a single or multi-pathogen transmission epidemiological scenario. Our results show that our method for integrating multiple types of data is powerful and that important enhancements to understanding of underlying infection dynamics can be made through the integration of just small amounts of genetic typing data.

## SUPPLEMENTARY MATERIAL

To view supplementary material for this article, please visit http://dx.doi.org/10.1017/S0031182016000044
